# Silymarin prevents acetaminophen-induced hepatotoxicity in mice

**DOI:** 10.1371/journal.pone.0191353

**Published:** 2018-01-17

**Authors:** Zuzana Papackova, Marie Heczkova, Helena Dankova, Eva Sticova, Alena Lodererova, Lenka Bartonova, Martin Poruba, Monika Cahova

**Affiliations:** 1 Department of Metabolism and Diabetes, Institute for Clinical and Experimental Medicine, Prague, Czech Republic; 2 Department of Veterinary Science, Faculty of Agrobiology, Food and Natural Resources, Czech University of Life Sciences Prague, Prague, Czech Republic; 3 Clinical and Transplant Pathology Department, Institute for Clinical and Experimental Medicine, Prague, Czech Republic; 4 Department of Pharmacology, Faculty of Medicine and Dentistry, Palacky University Olomouc, Olomouc, Czech Republic; Toho Daigaku, JAPAN

## Abstract

Acetaminophen or paracetamol (APAP) overdose is a common cause of liver injury. Silymarin (SLM) is a hepatoprotective agent widely used for treating liver injury of different origin. In order to evaluate the possible beneficial effects of SLM, Balb/c mice were pretreated with SLM (100 mg/kg b.wt. *per os*) once daily for three days. Two hours after the last SLM dose, the mice were administered APAP (300 mg/kg b.wt. i.p.) and killed 6 (T_6_), 12 (T_12_) and 24 (T_24_) hours later. SLM-treated mice exhibited a significant reduction in APAP-induced liver injury, assessed according to AST and ALT release and histological examination. SLM treatment significantly reduced superoxide production, as indicated by lower GSSG content, lower HO-1 induction, alleviated nitrosative stress, decreased p-JNK activation and direct measurement of mitochondrial superoxide production *in vitro*. SLM did not affect the APAP-induced decrease in CYP2E1 activity and expression during the first 12 hrs. Neutrophil infiltration and enhanced expression of inflammatory markers were first detected at T_12_ in both groups. Inflammation progressed in the APAP group at T_24_ but became attenuated in SLM-treated animals. Histological examination suggests that necrosis the dominant cell death pathway in APAP intoxication, which is partially preventable by SLM pretreatment. We demonstrate that SLM significantly protects against APAP-induced liver damage through the scavenger activity of SLM and the reduction of superoxide and peroxynitrite content. Neutrophil-induced damage is probably secondary to necrosis development.

## Introduction

Acetaminophen (N-acetyl-ρ-aminophen, APAP) is a safe and effective analgesic/antipyretic drug when used at therapeutic levels [1]. However, APAP overdose results in centrilobular hepatic necrosis, which can be fatal [2]. APAP toxicity is initiated by the formation of the reactive metabolite N-acetyl-p-benzoquinone imine (NAPQI). NAPQI is catalysed by cytochrome P450 CYP2E1, which is responsible for liver injury through the depletion of glutathione [3]. Once GSH is exhausted, any remaining NAPQI formed will react with alternative targets, in particular cellular proteins such as mitochondrial proteins, and induce mitochondrial oxidative stress and dysfunction [4]. Increased superoxide production is central to consecutive pathological processes. The spontaneous reaction of superoxide and nitric oxide gives rise to the generation of peroxynitrite. Knight et al. [5, 6] confirmed that peroxynitrite plays a critical role in the mechanisms of APAP-induced hepatotoxicity. Increased oxidant stress results in the activation of c-Jun N-terminal kinases (JNK) 1/2, which translocate to the mitochondria, eventually triggering the opening of the mitochondrial membrane permeability transition (MPT) pore [7] and releasing mitochondrial intermembrane proteins such as apoptosis-inducing factor (AIF). The translocation of these proteins to the nucleus causes nuclear DNA fragmentation [8]. The extensive mitochondrial dysfunction resulting in ATP depletion together with nuclear DNA damage leads to necrotic cell death [9]. Currently, APAP-induced liver injury is a popular mechanistic model for testing phytotherapeutic and other hepatoprotective interventions.

The use of natural products in the prevention and treatment of liver disease has gained considerable popularity [10]. Silymarin (SLM) is a polyphenolic component isolated from the fruits and seeds of the milk thistle plant *Silybum marianum* (Asteraceae family) [11]. Silymarin extract contains approximately 65% to 80% flavonolignans (silybin A, silybin B, isosilybin A, isosilybin B, silychristin and silydianin), a small proportion of flavonoids and approximately 20% to 35% fatty acids and polyphenolic compounds, which possess a range of metabolic regulatory effects [12].

The hepatoprotective properties of silymarin in APAP intoxication have been previously described [13–16]. Nevertheless, most of these studies only concentrate on the final effect of silymarin in terms of reducing death rate and not on the detailed mechanisms of its protective effects. Furthermore, a substantial number of studies have been performed on rats, which are an unsuitable model for APAP toxicity. In humans, APAP-induced liver injury involves mitochondrial damage, oxidative stress, c-Jun terminal kinase activation and nuclear DNA fragmentation. The mode of cell death is oncotic necrosis, a similar mechanism to the one that occurs during APAP intoxication in mice. However, rats develop low or no oxidative stress and thus no injury; hepatoma cells may develop injury but through a different mechanism to the mechanisms in mice and humans [17]. In this mouse model study, we examined the effect of silymarin on critical events during the initiation and progression of APAP hepatotoxicity, particularly CYP2E1 metabolism, superoxide production, oxidative and nitrosative stress, inflammation and apoptotic and necrotic cell death.

## Materials and methods

### Animals

Male BALB/c mice were kept in a temperature-controlled room under a 12:12 hour light-dark cycle. The animals had free access to drinking water and were fed a standard chow diet. All experiments were performed in agreement with the Animal Protection Law of the Czech Republic 311/1997, which is in compliance with the Principles of Laboratory Animal Care (NIH Guide to the Care and Use of Laboratory Animals, 8^th^ edition, 2013) and were approved by the ethical committee of the Institute for Clinical and Experimental Medicine.

### Experimental design

Fifty-six mice were randomly divided into 7 groups (n = 8): (1) vehicle control, (2) APAP 6 hours, (3) APAP 6 hours + SLM, (4) APAP 12 hours, (5) APAP 12 hours + SLM, (6) APAP 24 hours, (7) APAP 24 hours + SLM. The effect of SLM on CYP2E1 activity or expression and GSH/GSSG content was tested in separate group designated as SLM. Micronized silymarin was purchased from Favea s.r.o., Koprivnice, CR. The suspension of the silymarin and 25% xanthan gum in the appropriate dose was administered per os by intragastric gauche. Mice were pretreated with four doses (1 dose per day) of silymarin (100 mg/kg). The last dose was applied two hours prior to APAP administration. On the fourth day after fasting for 8 hours, mice were treated with APAP (300 mg/kg). APAP (Sigma Aldrich, St. Louis, MO USA) was dissolved in 0,6 ml of warm (37°C) sterile phosphate buffered saline (PBS) and injected intraperitoneally. Animals were killed 6 (T_6_), 12 (T_12_) and 24 (T_24_) hours after APAP administration, after which serum and liver tissue were collected.

### Hepatotoxicity assay

Plasma levels of alanine aminotransferase (ALT) and aspartate aminotransferase (AST) were measured using a commercially available kit (Sigma Aldrich, St. Louis, MO, USA) according to the manufacturer’s instructions.

### Histology and immunohistochemistry

Liver pieces were fixed in formalin and embedded in paraffin. Samples were then sectioned and stained with haematoxylin-eosin to further evaluate liver damage. Nitrotyrosine staining was performed according to Knight [5]. For detection of antigen three step visualization system was used: primary anti-nitrotyrosine antibody (MyBioSource, Inc., San Diego, CA, MBS 2001557, dilution 1:2000), biotinylated goat anti rabbit IgG (H+L), Vectastain Elite ABC Reagent (Vector Laboratories, Inc., Burlingame, CA, USA) and DakoLiquid DAB+ Substrate Chromogen System (Dako, Glostrup, Denmark).

### Total cytochrome P450 determination and CYP2E1 activity assay

The total cytochrome P450 concentration in liver homogenate and microsomes was determined spectrophotometrically using a method described by Guengerich et al. [18]. Cytochrome P450 concentration was calculated using a carbon monoxide difference spectrum between 450 and 490 nm for dithionite-reduced samples. CYP2E1 enzyme activity was determined using a method based on chlorzoxazone 6-hydroxylation [19]. In brief, 1 ml of the reaction mixture (100 mM phosphate buffer, pH 7.4, NADPH generating system, 2.5 mM chlorzoxazone) was incubated with liver homogenate or liver microsomes (160 pmol P450) for 20 min. The reaction was terminated by adding 50 μl of 42.5% phosphoric acid and 2 ml of a mixture of propan-2-ol/chloroform 15/85 (w/w). The mixture was centrifuged at 1000 g for 10 min, sediment was dried under nitrogen and the residue was dissolved in 200 μl of a mobile phase (0.5% acetic acid in 75% acetonitrile). 50 μl of the solution was injected into the Shimadzu HPLC system (LC Prominence, Shimadzu, Kyoto, Japan) at a constant flow rate of 1 ml/min and with UV detection at 287 nm.

### Isolation of liver mitochondria and preparation of submitochondrial particles

Liver mitochondria were prepared by differential centrifugation as described by Bustamante et al. [19] with some modifications. Liver tissue was homogenised at 0°C using a teflon-glass homogeniser as a 10% homogenate in a medium containing 220 mM mannitol, 70 mM sucrose and 1 mM HEPES, pH 7.2 (MSH medium). Crude impurities were removed by centrifugation at 800 g for 10 min and the remaining supernatant was centrifuged for 10 min at 8 000 g. The pelleted mitochondria were resuspended in the MSH medium, washed twice under 10-min centrifugation at 8 000 g and finally resuspended at a concentration of 20–30 mg protein/ml. Mitochondrial proteins were determined using the BCA method (Thermo Fisher Scientific, Waltham, MA, USA). Submitochondrial particles (SMP) were prepared according to Ide et al. [20]. Briefly, isolated mitochondria were sonicated and pelleted by centrifugation at 48 000 g for 10 min. The resultant pellet was washed three times in MSH buffer in order to get rid of matrix components and then stored at -80°C.

### Fluorometric determination of reactive oxygen species production

ROS production in SMPs *in vitro* was measured using a DCFDA (Cell Biolabs, San Diego, CA, USA) probe, as described previously [21]. Briefly, the assay was performed with approximately 0.2 mg of mitochondrial protein per ml in MAS buffer (70 mM sucrose, 220 mM mannitol, 10 mM KH_2_PO_4_, 5 mM MgCl_2_, 2 mM HEPES, 1 M EGTA, pH = 7.2). The measurement was performed either in basal MAS buffer only or in a medium supplemented with either 10 mM NADH -/+ 10 μM antimycin or 10 mM succinate -/+ 10 μM antimycin. The final concentration of DCFDA was 10 μM and the excitation/emission wavelength was 485/528 nm. The fluorescence signal rose linearly from 0 until the 45^th^ minute of the assay. The data presented were obtained 45 min after the start of the assay. All experiments were repeated in the absence of mitochondria, while background fluorescence changes were subtracted. The obtained values were normalised per mg of protein and expressed as a percentage of fluorescence under basal conditions (without substrates).

### Parameters of oxidative stress

Thiobarbituric acid-reactive substance (TBARS) content was determined using the ELISA TBARS determination kit (Exiqon, Woburn, MA, USA). Levels of reduced (GSH) and oxidised glutathione (GSSG) were assayed with the Glutathione in Whole Blood–HPLC detection kit (Chromsystems, Gräfelfing, Germany).

### Electrophoretic separation and immunodetection

Liver homogenate (20% wt/vol) was prepared using the Ultra-Turrax homogeniser (IKE, Worke, Staufen, Germany) in a homogenisation buffer (10 mM TRIS, 250 mM sucrose, 1 mM EDTA, 1 mM PMSF, 10 ug/ml leupeptin, 10 ug/ml aprotinin). Protein concentration was determined using the BCA method (Thermo Fisher Scientific, Waltham, MA, USA). Proteins were separated under denaturing conditions using SDS-PAGE and electroblotted to PVDF membranes. Phosphorylation of JNK was assessed by immunodetection using a specific antibody (Cell Signaling Technology, Danvers, MA, USA). The total expression of JNK was determined on the same membrane after striping and reblotting using a specific antibody (Cell Signaling Technology, Danvers, MA, USA). Bands were visualised by ELC and quantified using the FUJI LAS-3000 imager (FUJI FILM, Tokyo, Japan). Other proteins were quantified using specific antibodies: RIP-3 (Cell Signaling Technology, Danvers, MA, USA) and CYP2E1 (Abcam, Cambridge, UK). These membranes were exposed to medical X-Ray films and scanned using CanoScan Toolbox software, ver. 5.0. Medical X-ray films were analysed using ElfoMan software, ver. 2.6 (Semecky Inc., Prague, Czech Republic).

### Real-time RT-PCR

Liver samples were dissected and immediately frozen in liquid nitrogen. Total RNA was extracted using the Qiagen Mini RNeasy isolation kit (Qiagen, Hilden, Germany). A DNAase step was included to avoid possible DNA contamination. A standard amount of total RNA (1600 ng) was used to synthesise first-strand cDNA with the High Capacity RNA-to-cDNA Kit (Applied Biosystems, Foster City, CA, USA). The RT-PCR amplification mixture (25ul) contained 1 ul template cDNA, Syber Green master mix buffer (QuantiTect, Qiagen, Hilden, Germany) and 400nM (10 pmol/reaction) sense and antisense primer. The reaction was run on the ViiA 7 Real-Time PCR System (Thermo Fisher Scientific, USA). Results were analysed using SDS software, ver. 2.3 (Applied Biosystems, Foster City, CA, USA). The expression of genes of interest was normalised to the housekeeper gene (B2M) and calculated using the ΔΔCt method.

### Primer design

Primers were based upon known mice sequences available from the GeneBank Graphics database: *https://www.ncbi.nlm.nih.gov*. Primer design was performed with Primer3 software: http://www.frodo.wi.mit.edu (Table 1). CYP2E1 expression was assayed using Taqman primers/probes (Life Technologies, Carlsbad, CA, USA)

**Table 1:** Primer characteristics.

Gen	NCBI Ref. Sequence	Forward primer	Reverse primer
HO-1	NM_001199033.1	TGGGTCCTCACTCTCAGCTT	GTCGTGGTCAGTCAACATGG
Ccl2	NM_011333.3	ACTGAAGCCAGCTCTCTCTTCCTC	TTCCTTCTTGGGGTCAGCACAGAC
Ccr2	NM_009915.2	AGGAGCCATACCTGTAAATG	TTGATAGTATGCCGTGGATG
Ccl3	NM_011337.2	GCCCTTGCTGTTCTTCTCTGT	GGCAATCAGTTCCAGGTCAGT
TNFα	NM_001278601.1	CACGTCGTAGCAAACCAC	TGTCCCTTGAAGAGAACCTG
IL 1β	NM_008361.4	CCTCACAAGCAGAGCACAAG	AGAGGCAAGGAGGAAACACA
IL 12	NM_001303244.1	GTAACCAGAAAGGTGCGTTC	AAAAGCCAACCAAGCAGAAG
iNOS	NM_001313921.1	TGGGAATGGAGACTGTCCCAG	GGGATCTGAATGTGATGTTTG

### Statistical analysis

Data are presented as mean ± SEM. Statistical analysis was performed using the Kruskal-Wallis test with multiple comparisons (n = 7–8). Differences were considered statistically significant at the level of p<0.05.

## Results

### SLM alleviates APAP hepatotoxicity

**Fig 1:**
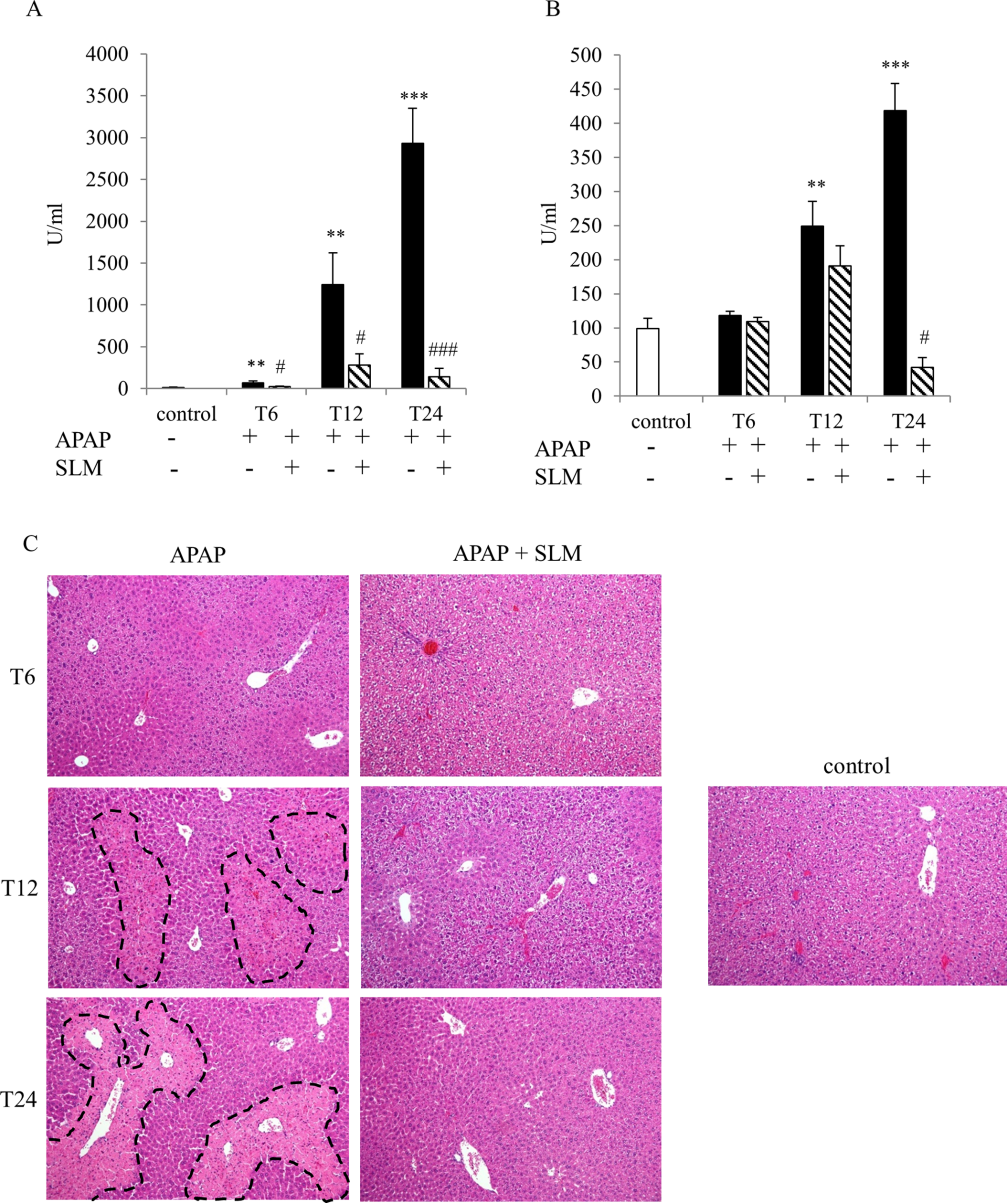
Effect of SLM on APAP-induced liver injury. (A) ALT and (B) AST serum content; (C) necrotic lesions (dashed lines) in the liver determined by hematoxylin and eosin staining, original magnification x 100. Data are presented as a mean ± SEM, n = 7. **p < 0.005, ***p < 0.001 APAP vs control; ^#^p < 0.05, ^###^p < 0.001 APAP+SLM vs APAP.

The hepatotoxic effect of APAP at a moderately toxic dose of 300 mg/kg b.wt. was confirmed by ALT and AST release into serum (Fig 1A and 1B). The first signs of liver injury were reflected by a rise in ALT serum content at T_6_. The developing liver injury is documented by elevated AST serum content at T_12_ and further exacerbated AST release at T_24_ as well as by growing elevation of ALT levels. In SLM-treated mice, all these parameters were significantly alleviated and had different dynamics. While in the APAP group, the liver injury had the continuous tendency to increase up to 24 hours post-APAP administration, in SLM-treated animals it peaked at T_12_.

**Fig 2:**
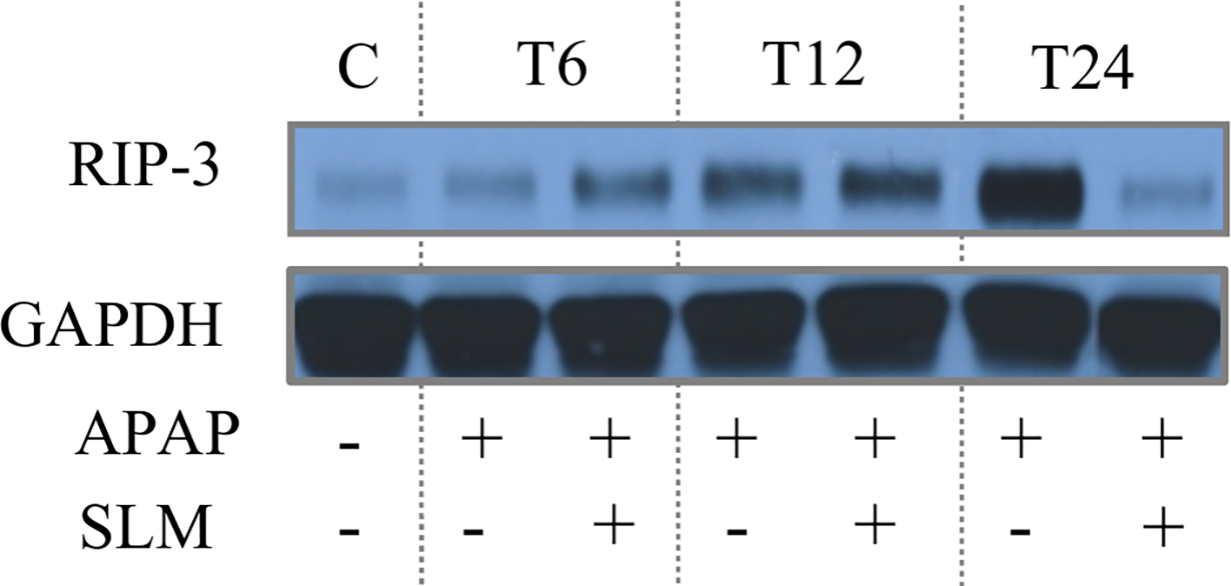
Effect of APAP and SLM on RIP-3 expression. The liver homogenate was separated by SDS-ELFO, immunoblotted and the proteins of interest were detected using specific antibody.

Histological examination of HE-stained tissue sections (Fig 1C) confirmed the signs of necrosis at T_12_ and severe necrosis at T_24_ in APAP groups. Receptor-interacting protein kinase (RIP-3) was recently discovered to be essential for some forms of necrosis [22]. APAP administration resulted in the increased expression of RIP3 and up to 12 hrs post administration this increase was not affected by SLM pretreatment (Fig 2). At T_24_ RIP3 expression in APAP group further increased but it returned to the control level in APAP+SLM group. Necrotic lesions were clearly visible in APAP-treated animals from T_12._

### SLM does not affect CYP2E1 expression or activity in the liver

**Fig 3:**
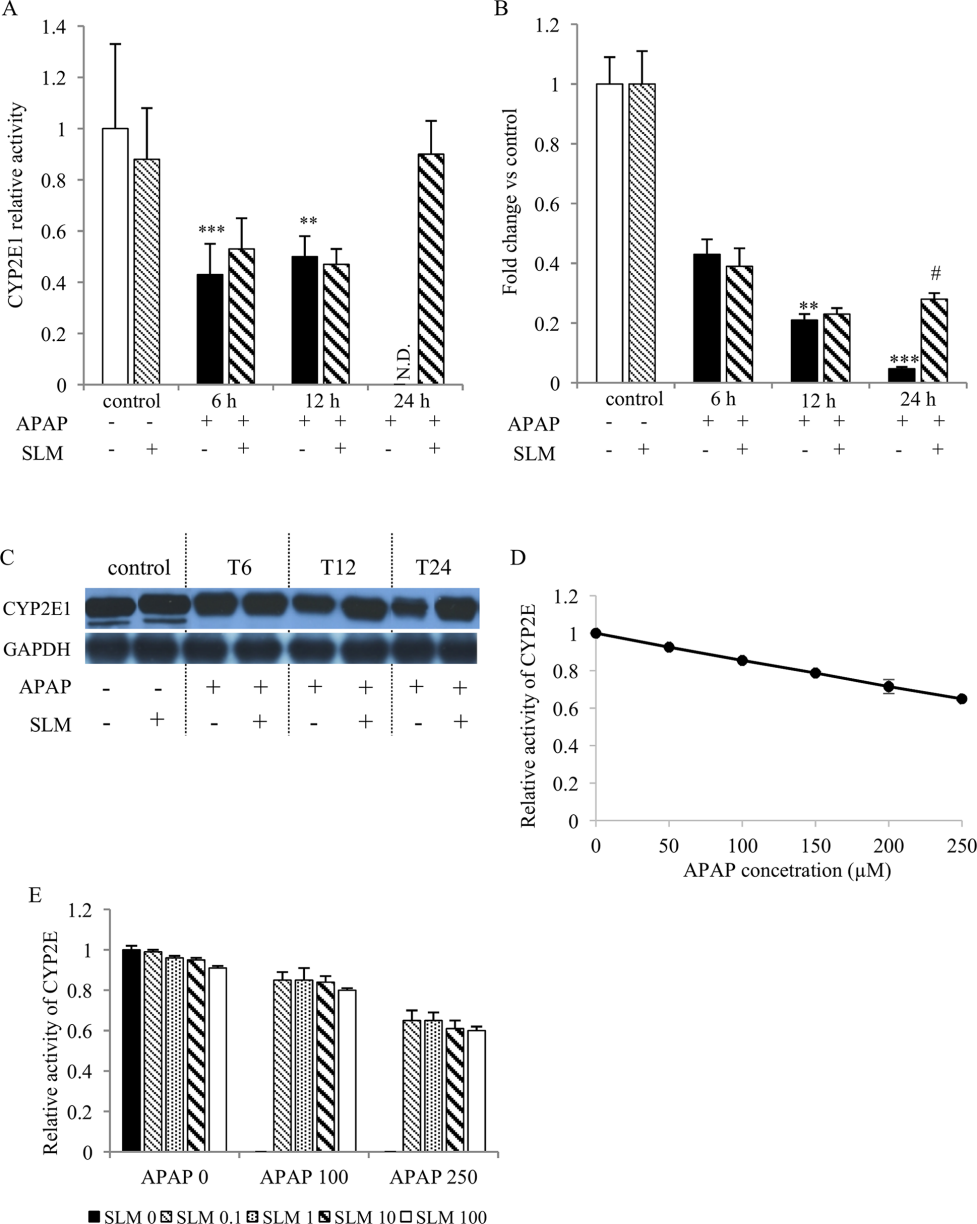
**Effect of APAP and SLM on CYP2E1 activity in vivo (A), mRNA expression (B), protein content (C) and CYPE1 activity in vitro (D, E).** Results represent mean ± SEM, n = 7. *p < 0.05, **p < 0.01, ***p < 0.001 APAP vs control. ^#^p < 0.05, ^##^p < 0.01 APAP + SLM vs APAP.

APAP is metabolised via the CYP2E1 isoform of cytochrome P450. CYP2E1 activity (Fig 3A) and mRNA expression (Fig 3B) in the liver were significantly reduced in APAP-administered animals at T_6_ and T_12_ irrespective of silymarin pretreatment. At T_24_, both CYP2E1 activity and mRNA expression were nearly undetectable in the APAP group, while the opposite trend was observed in SLM-treated mice where it reached half of the control values. CYP2E1 protein content (Fig 3C) was much less affected by the APAP treatment but we still observed a significant decrease from T_12_ onwards. SLM pretreatment alone had no effect on CYP2E1 activity or mRNA expression but it completely protected the CYP2E1 protein. In order to test the possible direct effects of APAP and SLM, we measured CYP2E1 activity in increasing APAP and SLM concentrations (Fig 3D and 3E). We observed a concentration-dependent inhibitory effect of APAP; however, even at its highest concentration (250 μM) CYP2E1 activity decreased only by 40%, which is much less than *in vivo*. SLM had no effect on APAP-dependent inhibition of CYP2E1 activity. In conclusion, our data indicate that APAP negatively affects CYP2E1 activity and SLM does not influence the interaction between CYP2E1 and APAP, at least in the early phases of APAP intoxication.

### SLM decreases APAP-induced reactive oxygen species formation from succinate

**Fig 4:**
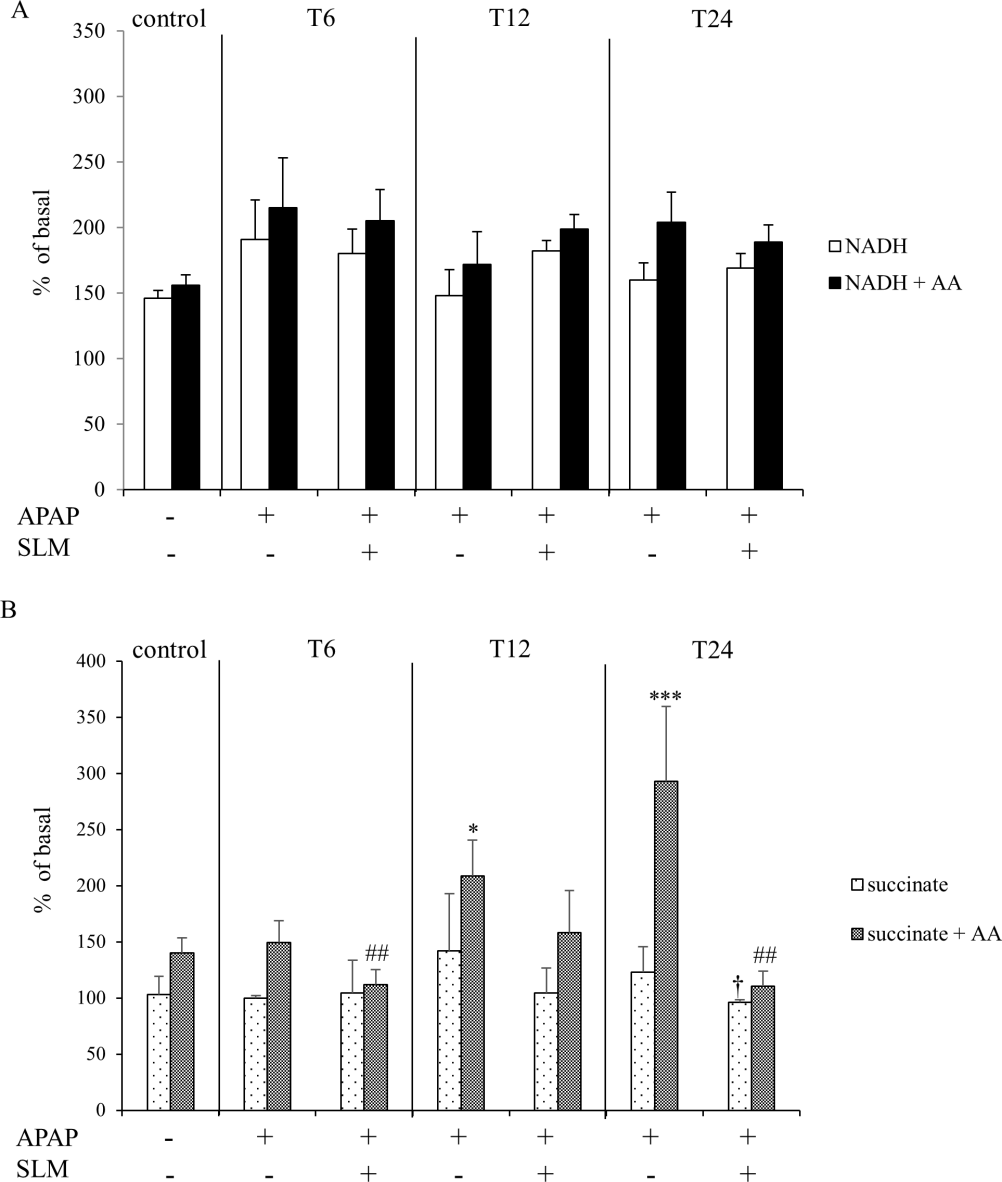
Effect of APAP and SLM on reactive oxygen species production from submitochondrial particles in vitro. (A) ROS production from NADH -/+ AA; (B) ROS production from succinate -/+ AA. Results are expressed as a percent of basal levels (without substrate). Data are presented as a mean ± SEM, n = 7. *p < 0.05, ***p < 0.001 APAP vs control (succinate + AA); ^†^ p < 0.05 APAP + SLM vs APAP (only succinate); ^#^p < 0.05, ^##^ p < 0.01 APAP + SLM vs APAP (succinate + AA).

Electron transport through the mitochondrial respiratory chain is associated with potential reactive oxygen species (ROS) generation, a risk that is significantly exacerbated when the functions of mitochondrial respiratory chain components are compromised. To characterise the effect of SLM treatment on APAP-induced ROS formation, we used an *in vitro* model of submitochondrial particles. ROS production was measured using a DCFDA fluorescent probe on two different substrates: (1) NADH as a source of electrons for complex I and (2) succinate for succinate dehydrogenase associated with complex II (Fig 4). We observed no effect, either of APAP or of SLM, on NADH-dependent ROS production from submitochondrial particles (Fig 4A). In contrast, when using succinate as a substrate we observed enhanced ROS production from submitochondrial particles in APAP-administered mice (T_12_ and T_24_), an effect that is substantially exacerbated in the presence of antimycin (Fig 4B). SLM attenuated ROS production with its effect most pronounced at T_24_. In conclusion, our data show that APAP increases ROS production, particularly from succinate dehydrogenase-dependent substrate, and that this phenomenon is alleviated by SLM pre-treatment.

### SLM diminishes oxidative and nitrosative stress

**Fig 5:**
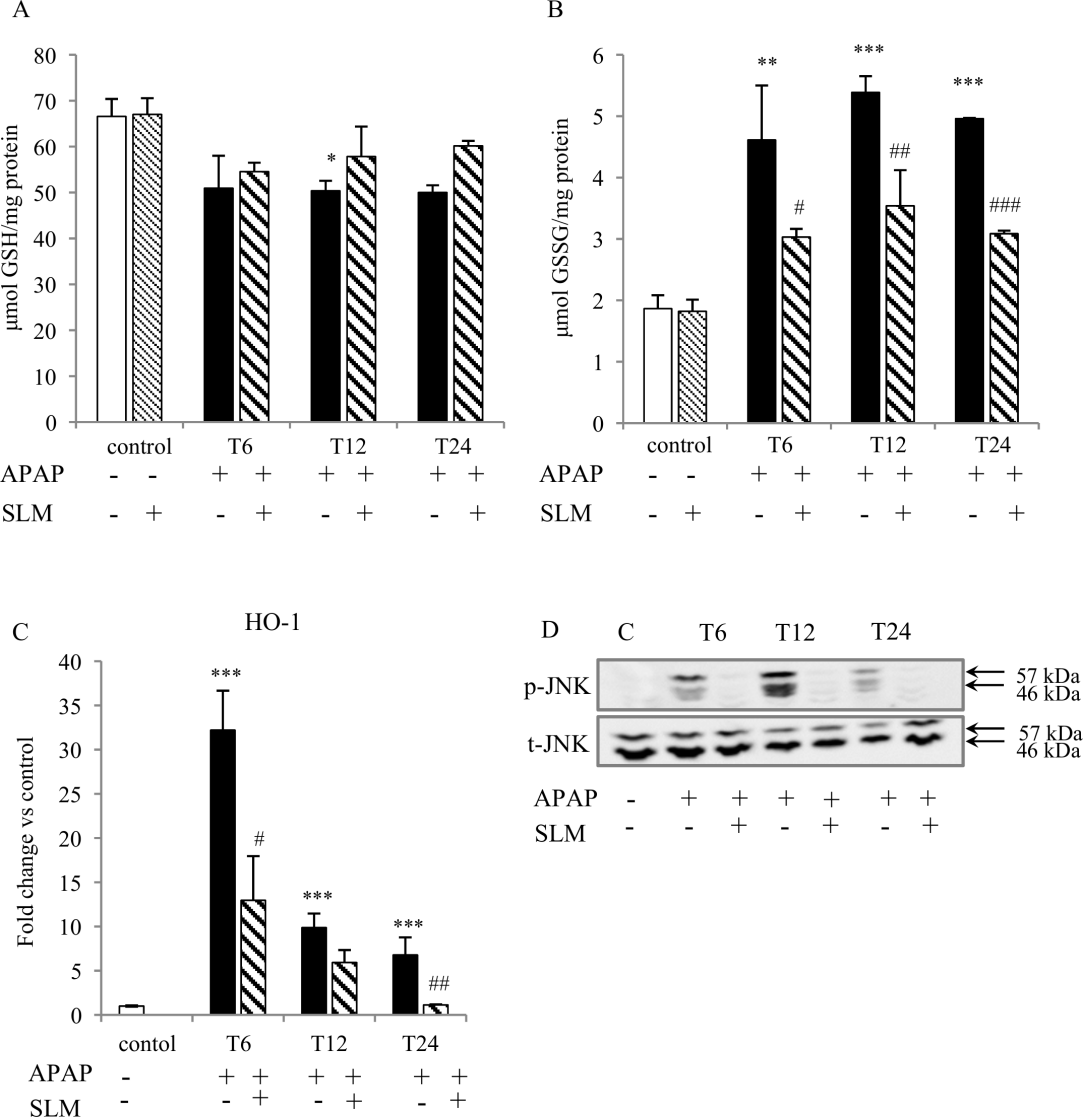
Effect of APAP and SLM on oxidative stress in the liver. (A) GSH content in liver homogenate; (B) GSSG content in liver homogenate; (C) HO-1 mRNA expression; (D) p-JNK and t-JNK protein expression in liver homogenate. Results are expressed as mean ± SEM, n = 7. *p < 0.05, **p < 0.01, ***p < 0.001 APAP group vs control; ^#^p < 0.05, ^##^ p < 0.01, ^###^ p < 0.001 APAP + SLM group vs APAP group.

**Fig 6:**
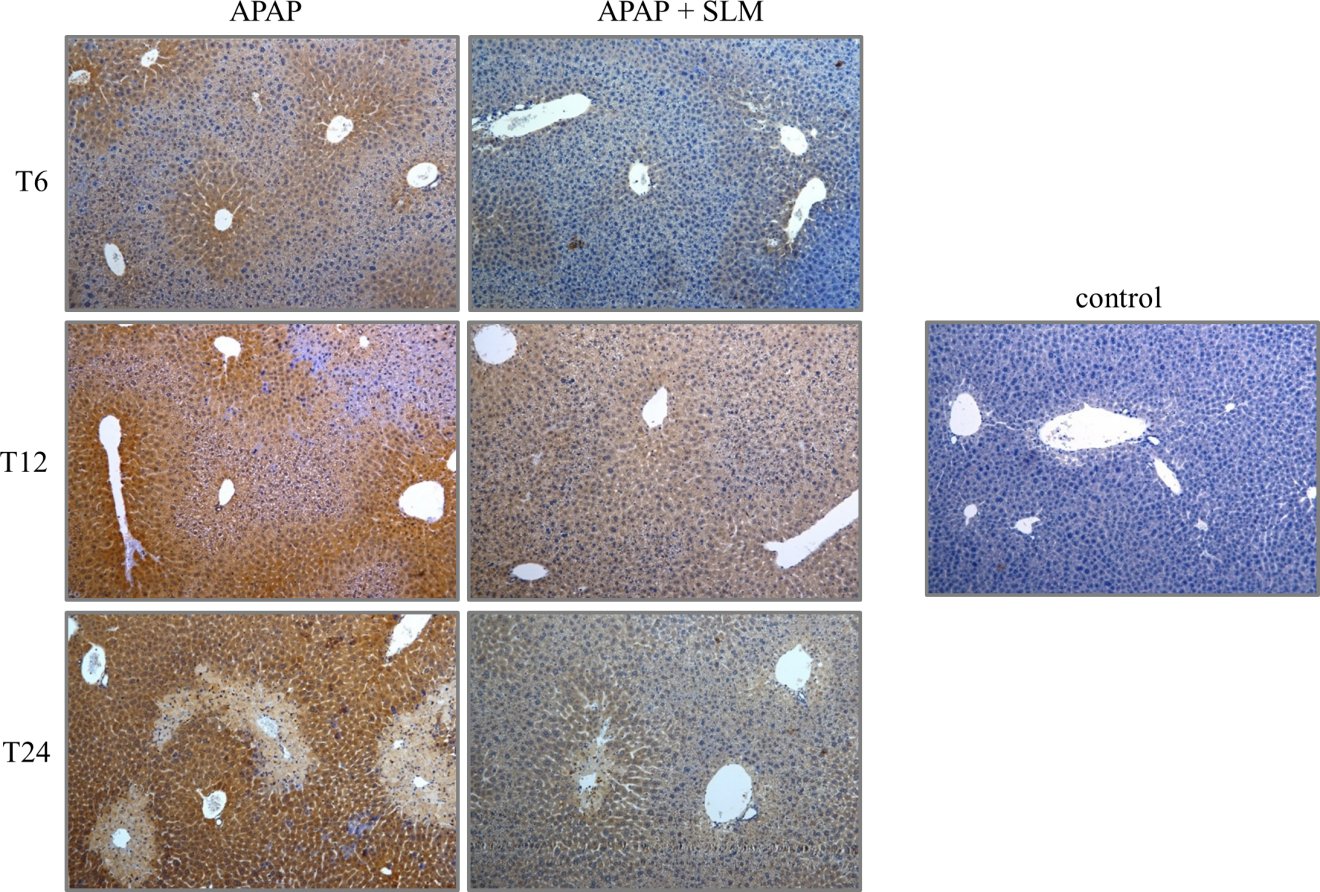
Nitrotyrosine formation in liver. Nitrotyrosine staining is shown as dark brown colored sections. Blue color identified healthy hepatocytes. Original magification x 100.

**Fig 7:**
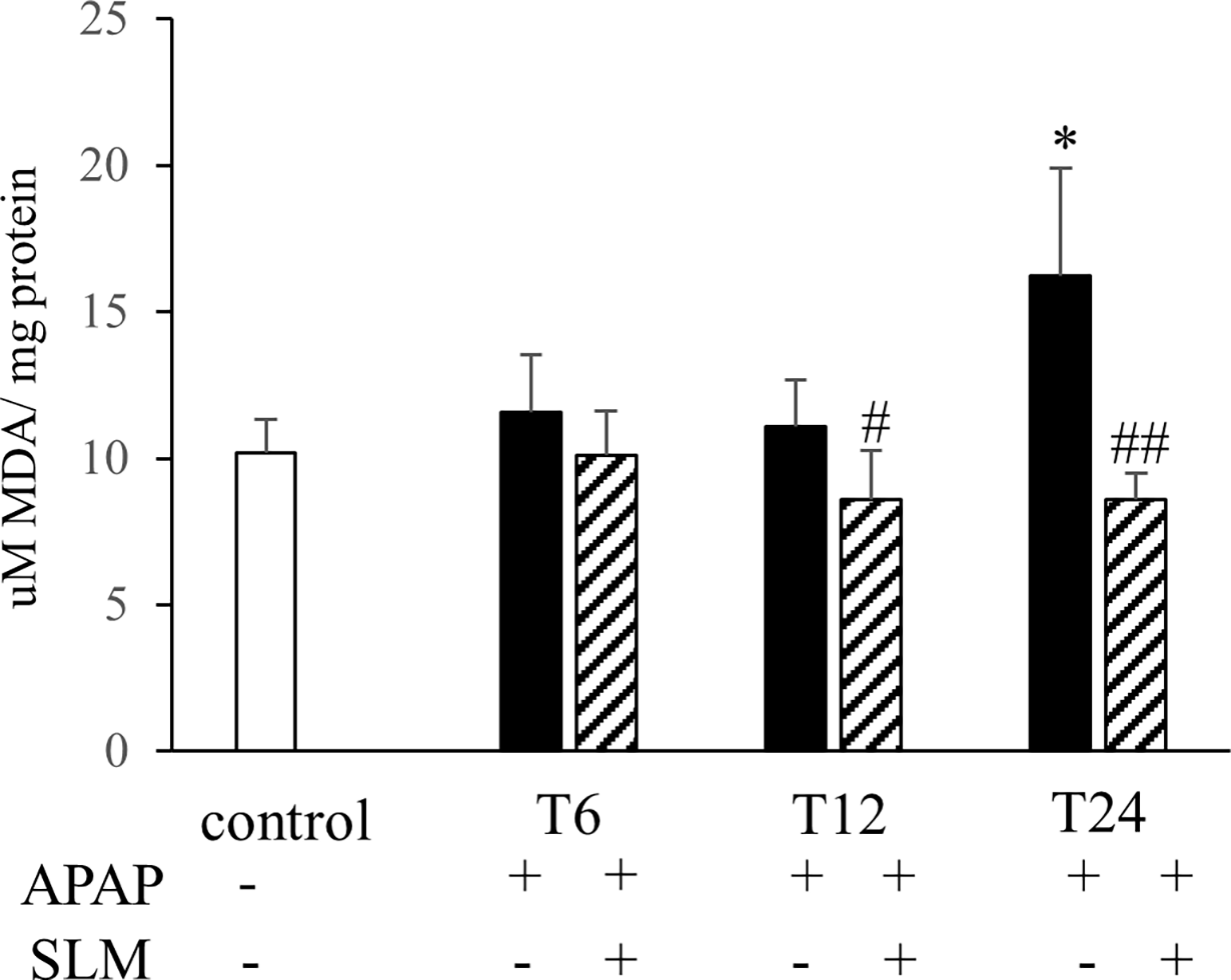
Effect of APAP and SLM on lipid peroxidation in the liver. Data are given as mean ± SEM, n = 7. *p < 0.05 APAP vs control; ^#^p < 0.05, ^##^p < 0.01 APAP + SLM vs APAP.

Increased production of ROS due to the compromised performance of mitochondria after APAP intoxication is considered the primary cause of liver function loss. In our experimental setting, several parameters indicated severe oxidative stress consequent to APAP administration. As expected, APAP administration resulted in a decrease in GSH content in all groups (Fig 5A). SLM-pretreated groups exhibited a tendency for less GSH depletion but this did not reach statistical significance. In contrast, APAP administration resulted in a significant increase in GSSG content, which was alleviated by silymarin at all time points (Fig 5B). Silymarin alone had no effect on GSH or GSSG content. HO-1 is a cytoprotective enzyme involved in the antioxidant defence. At T_6_, we observed more than 30-fold increase in HO-1 mRNA expression in the APAP group, but only a 10-fold increase in the APAP+SLM group. Along with the prolonged time from APAP administration, HO-1 expression exhibited a decreasing trend in all groups. Nevertheless, although normalised in APAP+SLM at T_24_, it remained elevated (8-fold) in the APAP group (Fig 5C). Because oxidative stress is understood to activate JNK kinase, we next measured the effect of SLM on JNK phosphorylation as a marker of activation. As shown in Fig 5D, APAP elicited significant JNK phosphorylation with a maximum at T_12_, which was attenuated in the presence of SLM. The presence of the highly reactive superoxide radical is difficult to measure directly *in vivo*, but it is possible to detect final compounds resulting from its interaction with biomolecules. Interaction between superoxide and NO results in the generation of ONOO^-^ radicals, which readily nitrate tyrosine residues in proteins to form nitrotyrosines [23]. We observed significant nitrotyrosine formation with a progressively rising tendency from T_6_ until T_24_ in all APAP-treated animals. Nitrotyrosine staining was weaker in the SLM-treated groups with a maximum at T_12_ but less intensive at T_24_ (Fig 6). MDA is a final product of lipid peroxidation resulting from a reaction between plasma phospholipids and the hydroxyl radical. In contrast to the rapid onset of nitrotyrosine formation, MDA content was not elevated compared with controls until T_24_ in APAP-administered animals. The protective effect of SLM was first detected at T_12_ and became fully apparent at T_24_ (Fig 7). Taken together, these data indicate that APAP-administered animals experience high exposition to reactive oxygen species in the liver and that the effect is significantly lower in the presence of SLM. Furthermore, we deduce that lipids seem to be less relevant targets of oxidative stress than proteins in APAP-induced hepatotoxicity.

### SLM reduces late-onset inflammation

**Fig 8:**
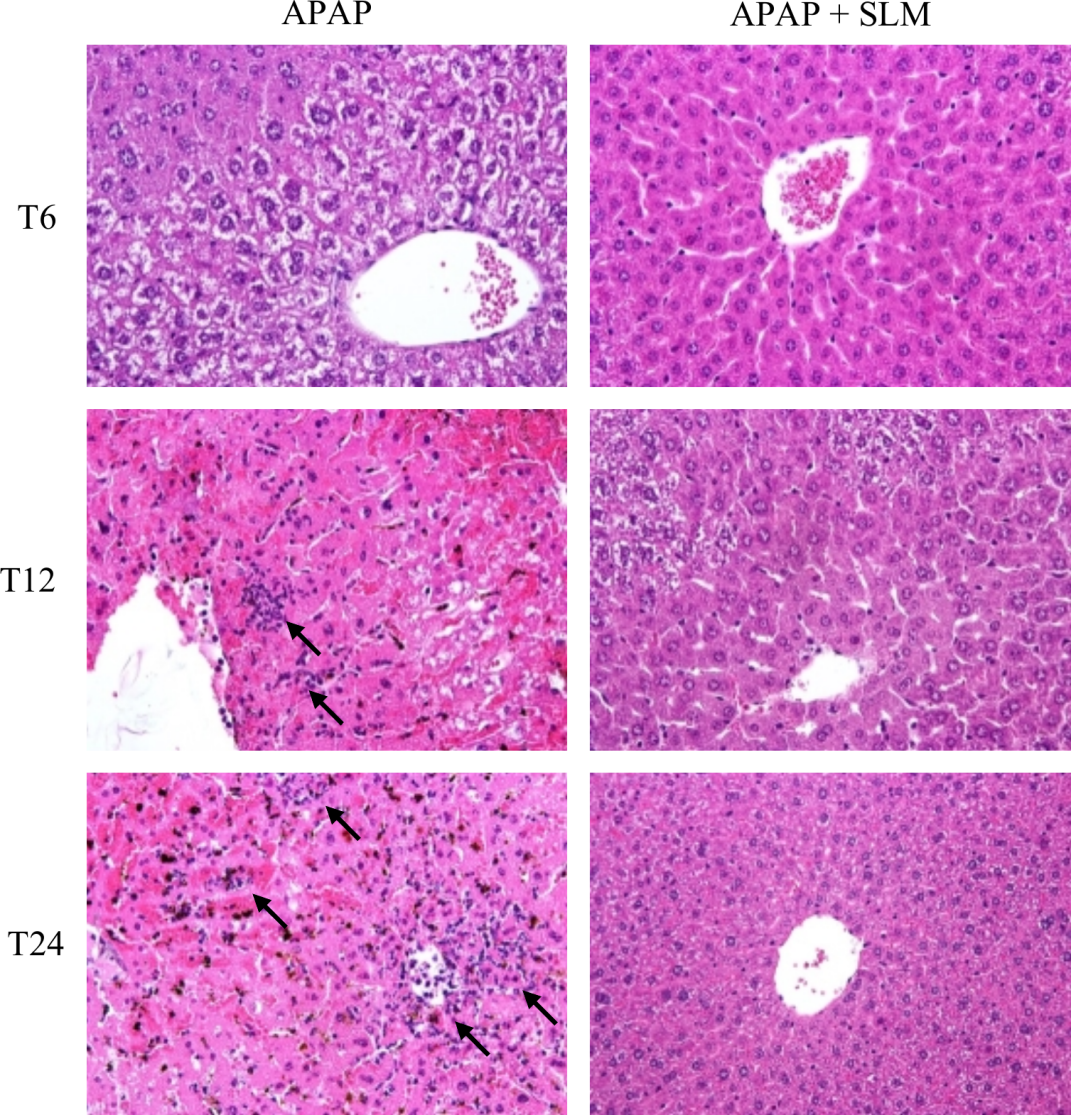
Effect of APAP and SLM on neutrophil infiltration. Tissue sections were stained with hematoxylin and eosin, black arrows indicate infiltrating neutrophiles. Original magnification x 600.

**Fig 9:**
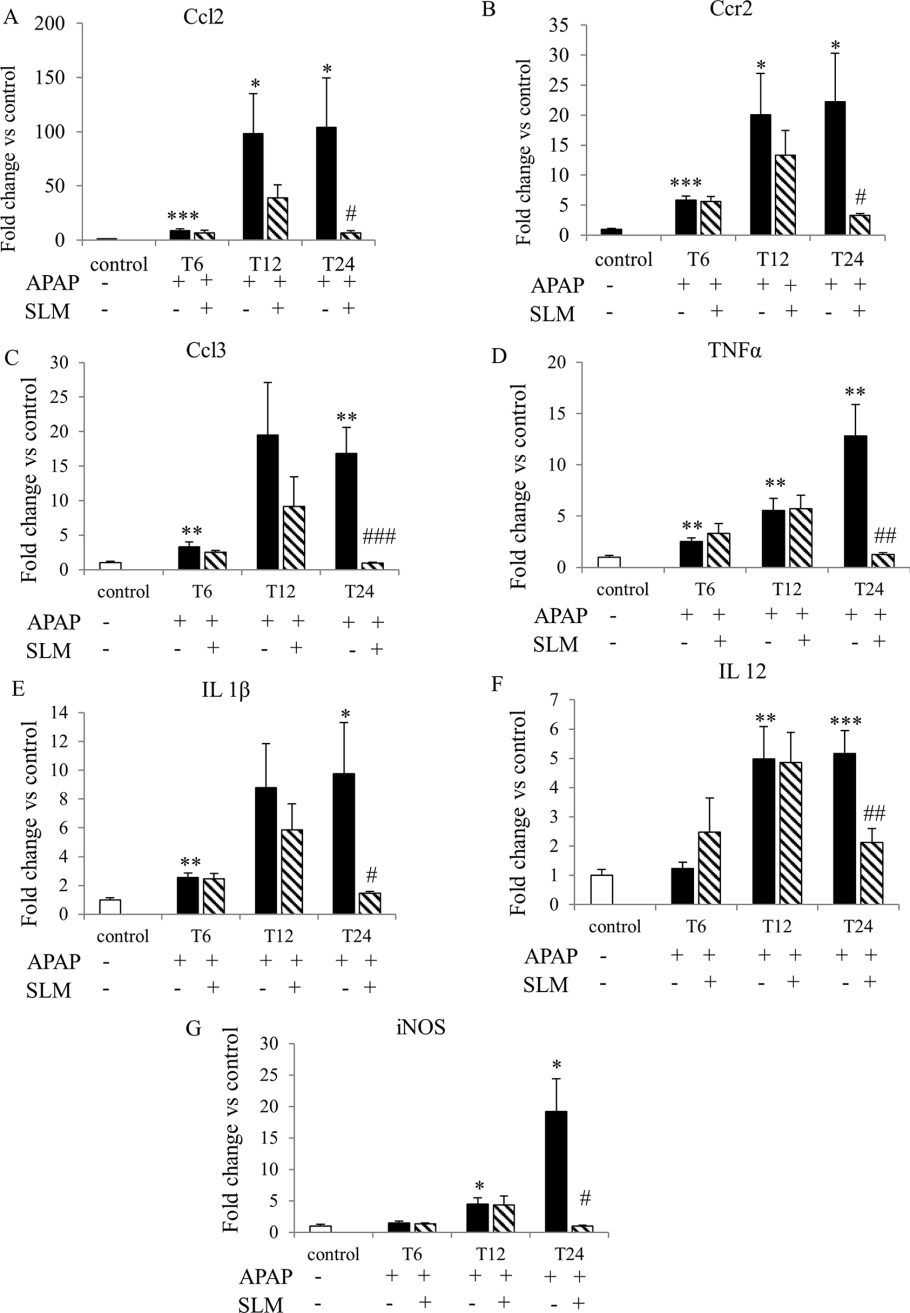
Effect of APAP and SLM on mRNA expression of selected inflammatory markers. (A) Ccl2; (B) Ccr2; (C) Ccl3; (D) TNFα; (E) IL 1β; (F) IL 12; (G) iNOS. Data are expressed as a fold change related to the untreated (control) group. Values are given as a mean ± SEM, n = 7. *p < 0.05, **p < 0.01, ***p < 0.001 APAP vs control; ^#^p < 0.05, ^##^p < 0.01, ^###^p < 0.001 APAP+SLM vs APAP.

Histological analysis revealed that inflammatory infiltrate accompanied necrosis in the livers of the APAP and APAP+SLM groups at T_12_ and at T_24_. Infiltrating neutrophils were observed in the APAP group but the infiltration was suppressed in the APAP+SLM group (Fig 8). The increased expression of chemokine CCl2 and its corresponding receptor CCr2 was detectable at T_6_, which was comparable for both the APAP and APAP+SLM groups. At T_12_, a marked difference in expression was observed between the SLM-pretreated and non-treated animals, the expression being significantly higher in the latter. At T_24_, the expression of both inflammatory markers remained high in the APAP group but returned back to almost control values in the APAP+SLM group (Fig 9A and 9B). We observed similar dynamics in the case of chemokine CCl3, except that expression was decreased in both of these groups at T_24_ (Fig 9C). Expression of TNFα, IL1-β and IL-12 reached a maximum at T_12_, which was comparable for both the APAP and APAP+SLM groups (Fig 9D, 9E and 9F). In contrast, at T_24_, expression was almost normalised in APAP+SLM mice but remained high in APAP-only treated animals. iNOS catalyses the formation of NO in macrophages as part of the immune defence mechanism, as NO is a free radical with an unpaired electron. iNOS expression was elevated (approximately 5-fold) at T_12_ in both the APAP and APAP+SLM groups (Fig 9G). While in APAP only-treated animals expression continued rising up to 20-fold at T_24_, in the APAP+SLM group it was completely normalised 24 hours after APAP administration. Our data confirm the development of inflammatory changes in the liver with the onset occurring 12 hours after APAP administration. The intensity of the inflammatory response was significantly alleviated in the APAP+SLM group and, in contrast to APAP only-treated animals, was not propagated at T_24_.

## Discussion

We provide evidence that preventive treatment with silymarin significantly alleviates APAP-induced liver injury in mice. The major objective of this study was to evaluate the potential beneficial effect of silymarin on APAP-induced hepatotoxicity. We described the effect of silymarin on different aspects of APAP-induced liver injury, particularly CYP2E1 metabolism, oxidative and nitrosative stress and inflammation. We identified that the critical actions of silymarin are scavenging of radical forms of oxygen and prevention of peroxynitrite formation. We confirmed that the protective effect of silymarin is to eliminate consecutive pathological events, such as increased ALT and AST levels, inflammation and necrosis.

The hepatotoxicity of APAP begins with the metabolic conversion of APAP to its reactive metabolite, N-acetyl-p-benzoquinone imine (NAPQI), which is catabolised by CYP2E1 [24–26]. In this context, Lee et al. [27] demonstrated resistance to APAP hepatotoxicity in CYP2E1 knockout mice. Hypothetically, the protective effect of SLM on APAP-induced hepatotoxicity may be mediated by its ability to modulate the activity of CYP2E1. We and other authors show that APAP itself *in vivo* decreases the activity of CYP2E1, which may act as the protective mechanism against the formation of toxic NAPQI [28]. The data regarding the effect of SLM on CYP2E1 activity are inconsistent. Al-Rasheed et al. [29] reported a stimulatory effect of SLM on CYP2E1 activity in rats exposed to CCl4. In contrast, Miquez et al. [30] found no evidence for the interaction of silymarin with cytochrome P450 2E1 *in vitro*. However, the addition of these compounds counteracted allyl alcohol toxicity, associated lipid peroxidation and GSH depletion in isolated rat hepatocytes. We did not observed any effect of silymarin alone on CYP2E1 activity either *in vivo* or *in vitro*. We found that APAP decreased CYP2E1 activity by approximately 50% as early as 6 hours after APAP administration, an effect that lasted at least until T_12_. SLM treatment seemed to have no effect on the APAP-induced decrease of CYP2E1 activity. A significant difference between SLM-treated and untreated animals became apparent at T_24_ with literally no activity in the APAP group, in contrast to almost normal activity in the SLM-treated group. We explain the positive trend in the SLM-treated group as a marker of liver cell regeneration rather than as the direct effect of silymarin.

Current experimental evidence indicates that mitochondrial dysfunction and excessive ROS production resulting in severe oxidant stress are central to the intracellular mechanisms of APAP-induced injury in hepatocytes [31, 32]. Based on this study, several pieces of indirect evidence indicate that SLM-treated mice experience much less oxidative stress than untreated mice. First, liver content in the oxidised form of glutathione (GSSG) was significantly lower in SLM-treated mice at all time points, which suggests diminished ROS burden. Second, we observed a significant increase in HO-1 expression 6 hours after APAP intoxication. However, this upregulation was significantly less pronounced in SLM-treated mice (30-fold vs. 10-fold in controls). HO-1 is one of the most critical cytoprotective mechanisms activated during cellular stress [33]. Its expression is mainly regulated at the transcriptional level by Nrf2 in response to enhanced ROS production [34]. Therefore, the weaker activation in SLM-treated animals may reflect reduced ROS formation. Third, we observed significantly reduced nitrotyrosine content in the livers of APAP+SLM-treated animals compared with the APAP group. Tyrosine residues in cellular proteins can be nitrated by ONOO^-^ to form 3-nitrotyrosine, which is the most commonly used ONOO^-^ biomarker in biological systems [23]. ONOO^-^ is produced during the reaction of superoxide with NO and nitrotyrosines and thus indirectly reflects superoxide formation intensity. Finally, we observed significant attenuation of JNK kinase phosphorylation, which also indicates weaker oxidative stress. Taken together, these data suggest that in SLM-treated animals less ROS is available for reactions with biomolecules and subsequent injury.

The reduced ROS abundance may result from either decreased ROS production, enhanced ROS deactivation or a combination of both. In this study, we observed enhanced ROS production from submitochondrial particles prepared from the livers of APAP-treated mice when using succinate, but not NADH, as the substrate was effectively attenuated by silymarin treatment. Based on our experiments, although we cannot confirm decreased ROS production in SLM-treated animals *in vivo*, our *in vitro* data support this possibility. Another significant source of ROS may be iNOS in activated resident macrophages or infiltrating neutrophils. iNOS catalyses the production of nitric oxide from L-arginine and, in an oxidative environment, high levels of NO react with superoxide to form peroxynitrite. Nevertheless, in our experimental setting we did not observe an increase in iNOS expression until T_12_, while a significant amount of nitrotyrosine in the centrilobular region of the liver was detected as early as at T_6_. Opinions concerning the role of iNOS in APAP-induced hepatotoxicity are contradictory. Gardner et al. [35] showed that iNOS deficiency in mice is associated with decreased APAP hepatotoxicity, but this finding was not confirmed by an independent study [36]. Similarly, inconsistent results have been obtained using iNOS inhibitors [35, 37]. Because of the time discrepancy between the onset of massive upregulation of iNOS expression and the formation of nitrosylated proteins, we do not consider activated immune cell-derived ROS formation as the primary cause of APAP hepatotoxicity. Antioxidant properties of SLM have been reported in various experimental settings *in vivo*, such as secondary biliary cirrhosis [38], doxorubicin-induced oxidative stress [39] and in a rodent model of NASH [40]. Furthermore, silymarin is an effective antioxidant according to different *in vitro* assays, contributing to total antioxidant activity, reduced power, superoxide radical scavenging, hydrogen peroxide scavenging and metal chelating activities [41]. Based on these findings, we presume that a substantial part of the protective effect of silymarin is the reduction of the ROS load either due to the direct scavenging activity of silymarin or due to the attenuation of production from mitochondria.

Administration of APAP to experimental animals results in the accumulation of activated macrophages in centrilobular regions of the liver [42, 43] and in extensive sterile inflammation, but their roles in the pathogenesis of APAP-induced liver injury remain debatable [44]. Although one line of evidence shows that depletion of neutrophils/macrophages prior to APAP administration alleviates APAP-induced hepatotoxicity, a number of other studies have found no evidence for decreased neutrophil activation [45–47]. In our study, we first detected upregulated expression of inflammatory cytokines, which indicates the recruitment of activated monocytes at T_6_ after APAP administration regardless of SLM treatment. While in the APAP group inflammatory cytokine expression continued to increase at T_12_ and remained at the same level at T_24_, in SLM-treated mice the rise at T_12_ was less pronounced and returned close to normal at T_24_. According to the timeline, the most significant upregulation of inflammatory marker expression correlated with the onset and extent of necrotic lesions. Sterile inflammation is critically dependent on the release of damage-associated molecular patterns (DAMPs) from necrotic cells. Thus, a reduction in cell necrosis would attenuate DAMPs release and consequently reduce pro-inflammatory cytokine formation and neutrophil infiltration [48].

Despite the evidence for apoptotic signalling, necrosis is considered to be the main cell death pathway in the liver after APAP intoxication [49]. Necrosis used to be described as an acute and uncontrollable event caused by overwhelming physical or chemical trauma, but recently it has been reported that necrosis may occur in a regulated manner [50]. RIP family includes a group of Ser/Thr kinases that sense various stress signals and modulate cell survival and death. One particular member of this family, RIP3, was demonstrated to be required for necrosis in different cell types [22, 51, 52]. RIP3-mediated necrosis may be induced by a variety of death stimuli like death receptor ligands (TNFα, Fas ligand, TRAIL) or pathogens (viruses) [53]. Pathogen-associated molecular patterns (PAMPs) bind to pathogen-recognizing receptors (PRRs) and trigger necrotic death cascade. Importantly, endogenous molecules released from cells dying via necrosis (DAMPs) can bind to these PRRs, trigger sterile inflammation, and contribute to the further propagation of necrosis. This mechanism may be particularly important in APAP-induced liver injury when intracellular content is being released from the injured parenchymal cells.

Our data show a continuous increase in RIP3 expression in APAP-treated mice up to 24 hrs post APAP administration. This observation is in accordance with those of Ramachandran et al. who suggested RIP3 as a mediator of APAP hepatotoxicity [54]. In contrast to our experimental design, they followed the development of liver injury only until 6 hours post-APAP so we cannot compare the dynamics of RIP3 expression in both experiments. RIP3 abundance was similar in APAP and APAP+SLM groups at T_6_ and T_12_ but we found significant decrease of RIP3 expression in APAP+SLM vs APAP group at T_24_. This dynamics parallel the pattern of other markers of liver injury in APAP and APAP+SLM groups including serum AST and ALT levels and necrosis development.

Concerning the mechanism, how RIP3 may contribute to the liver injury, Zheng et al. formulated the hypothesis that RIP3 might increase energy metabolism-associated ROS production [22]. They demonstrated that RIP3 by still unknown process stimulates ROS production generated at ubisemiquinone site of mETT and this process has a key role in TNF cytotoxicity in NIH 3T3 cells. We demonstrated the increased ROS production from complex II-dependent substrate *in vitro* and both processes followed similar time course, i.e. gradual increase from 6 till 24 hrs post-APAP. Nevertheless, we cannot decide whether these two phenomena (RIP3 expression and ROS production) are in causative relationship or whether it is just co-incidence.

In this context, it is necessary to mention the study by Dara et al. who failed to detect RIP3 after APAP treatment in primary mouse hepatocytes (PHM) [55]. We propose that the discrepancy might be explained by the different experimental setting. RIP3 is very short-lived protein (half-life approx. 2.3 hrs, [56]) and PHM rapidly dedifferentiate in culture and thus quickly lose many hepatic proteins. Yang et al. reported that levels of RIPK3 protein in PHMs markedly decreased after 6 hrs in culture [57]. On the other hand, it was reported that RIPK3 is expressed in non-parenchymal liver cells like activated Kupffer cells, liver leukocytes and sinusoidal endothelial cells [56]. Therefore, we cannot exclude the possibility that RIPK3 we have detected in the liver homogenate originates from other than liver parenchymal cells. In this case, RIPK3 expression could be associated with immune response to liver injury.

In summary, this study demonstrates that SLM pretreatment significantly protects against APAP-induced liver damage. The main protective effects seem to be direct scavenging of superoxide by silymarin and attenuated ROS formation in the mitochondria, which result in reduced peroxynitrite formation. The reduced release of DAMPs may mitigate late-stage sterile inflammation, thus representing an additional mechanism of silymarin action. We found no evidence supporting the direct effect of silymarin on CYP2E1 activity.

## Supporting information

S1 Checklist NC3Rs ARRIVE guidelines checklist. (PDF)
